# Diverse roles of bitter melon (*Momordica charantia*) in prevention of oral cancer

**DOI:** 10.20517/2394-4722.2020.126

**Published:** 2021-03-09

**Authors:** Subhayan Sur, Ratna B. Ray

**Affiliations:** 1 Department of Pathology, Saint Louis University, St. Louis, MO 63104, USA.; 2 Cancer Center, Saint Louis University, St. Louis, MO 63104, USA.

**Keywords:** Bitter melon (*Momordica charantia*), oral squamous cell carcinoma, signal transduction, cancer metabolism, immune system, cancer prevention

## Abstract

Oral squamous cell carcinoma (OSCC) is one of the common lethal malignancies which is increasing rapidly in the world. Increasing risks from alcohol and tobacco habits, lack of early detection markers, lack of effective chemotherapeutic agents, recurrence and distant metastasis make the disease more complicated to manage. Laboratory-based studies and epidemiological studies indicate important roles of nutraceuticals to manage different cancers. The plant bitter melon (*Momordica charantia*) is a good source of nutrients and bio-active phytochemicals such as triterpenoids, triterpene glycosides, phenolic acids, flavonoids, lectins, sterols and proteins. The plant is widely grown in Asia, Africa, and South America. Bitter melon has traditionally been used as a folk medicine and Ayurvedic medicine in Asian culture to treat diseases such as diabetes, since ancient times. The crude extract and some of the isolated pure compounds of bitter melon show potential anticancer effects against different cancers. In this review, we shed light on its effect on OSCC. Bitter melon extract has been found to inhibit cell proliferation and metabolism, induce cell death and enhance the immune defense system in the prevention of OSCC *in vitro* and *in vivo*. Thus, bitter melon may be used as an attractive chemopreventive agent in progression towards OSCC clinical study.

## INTRODUCTION

Cancer is a genetic disease that involves uncontrolled cell proliferation due to the dynamic changes in the genome, and it is the second leading cause of death worldwide^[1–3]^. At the molecular level, cancer is highly heterogeneous with more than 200 distinct disease entities in humans due to the differences in initiating cells, pattern of acquired somatic mutations, alteration in molecular signaling events and influence of local tissue microenvironments^[4]^. Oral squamous cell carcinoma (OSCC) is the sixth most common cancer, which arises from the epithelium of the anterior tongue, cheek, floor of mouth, retro molar area, gingiva and the buccal mucosa. OSCC accounts for more than 90% of head and neck cancers^[5,6]^. The incidence of oral cancer continues to increase worldwide. A global estimate of lip and oral cavity cancer cases in 2018 was 354,864, which was 2% of all new cancer cases with 177,000 deaths every year^[7,8]^. In the USA, the estimated incidence rate of cancer of the oral cavity and pharynx in 2020 was 53,260, leading to 10,750 deaths^[9]^. Tobacco and alcohol use with synergistic effects, chewing betel quid and gutka (chewing tobacco) and human papillomavirus (HPV) infection are major risk factors for OSCC^[10]^. Additionally, genetic factors and age also influence OSCC development^[11]^.

OSCC develops from single neoplastic cells or stem cells towards malignancy through sequential changes of histopathology ranging from hyperplasia, dysplasia and carcinoma *in situ* to invasive carcinoma. The multistep carcinogenesis process is regulated by a series of genetic and epigenetic events, resulting in activation of oncogenes and inactivation of tumor suppressor genes, modulation of the tumor microenvironment and the immune system, epithelial-to-mesenchymal transition, and increased metabolism and energy production^[11–14]^. Despite improvement in surgical techniques, chemotherapy and radiation therapy, the overall survival rates are 40%−50%, and they have not been improved in the last few decades^[8]^. OSCC is a very aggressive tumor, and many patients are diagnosed when the disease is at locoregionally advanced stages. Also, lack of early detection markers, frequent association with metastasis and inadequacy of effective therapeutic regimens make the disease more complicated to manage^[15–17]^. However, targeted therapy and immune therapy show promising results to some extent. The FDA (Food & Drug Administration of the USA) approved EGFR targeting therapies that showed clinical activity in OSCC but achieved limited success^[18,19]^. The use of PD-1 and PD-L1 immune checkpoint inhibitors also showed promising results in a phase III clinical trial^[8]^. Other challenges in OSCC treatment are side effects, overtreatment, worsening prognosis and increasing treatment costs^[8]^. Thus, there is a critical clinical need to understand the disease process and to identify better therapeutic strategies for successfully managing the disease.

Medicinal values of different natural phytochemicals have been identified since ancient times, and around 80% of the world’s population still rely on plant products for primary health benefits^[20]^. Many registered drugs such as anthracyclines (doxorubicin, epirubicin), taxanes (paclitaxel, docetaxel), vinca alkaloids (vinblastine, vinorelbine), podophyllotoxin and its derivations have already been derived from different plant sources^[21]^. In case of cancer prevention, many phytochemicals show promising results in epidemiological studies and pre-clinical experiments, and some of those are already in clinical trials^[22]^.

Bitter melon *(Momordica charantia)* or bitter gourd, balsam pear, or karela, has been known for ages for the treatment of diabetes^[23]^. The plant belongs to the family Cucurbitaceae and grows in tropical and sub-tropical regions of Asia, Africa and South America. The crude extract or isolated compounds also show anti-lipidemic, antibacterial, antifungal and anti-HIV activities^[22,24,25]^. Potential anticancer activity of bitter melon crude extract (BME) and isolated compounds are reported in various *in vitro* and *in vivo* models^[26]^. These observations are quite promising in the prevention of multiple cancer types such as cancers of the skin, brain, oral tissues (head and neck), breast, lung, liver, stomach, colon, pancreas, kidney, uterine cervix, ovary, prostate and blood^[26]^. In the present review, we focus only on oral cancer and summarize the effect of bitter melon with molecular details. The main objective of this review is to make people more familiar with bitter melon for its health benefits, especially to prevent oral cancer for successful management of this deadly disease.

## NUTRITIONAL VALUE OF BITTER MELON

**Figure 1.**
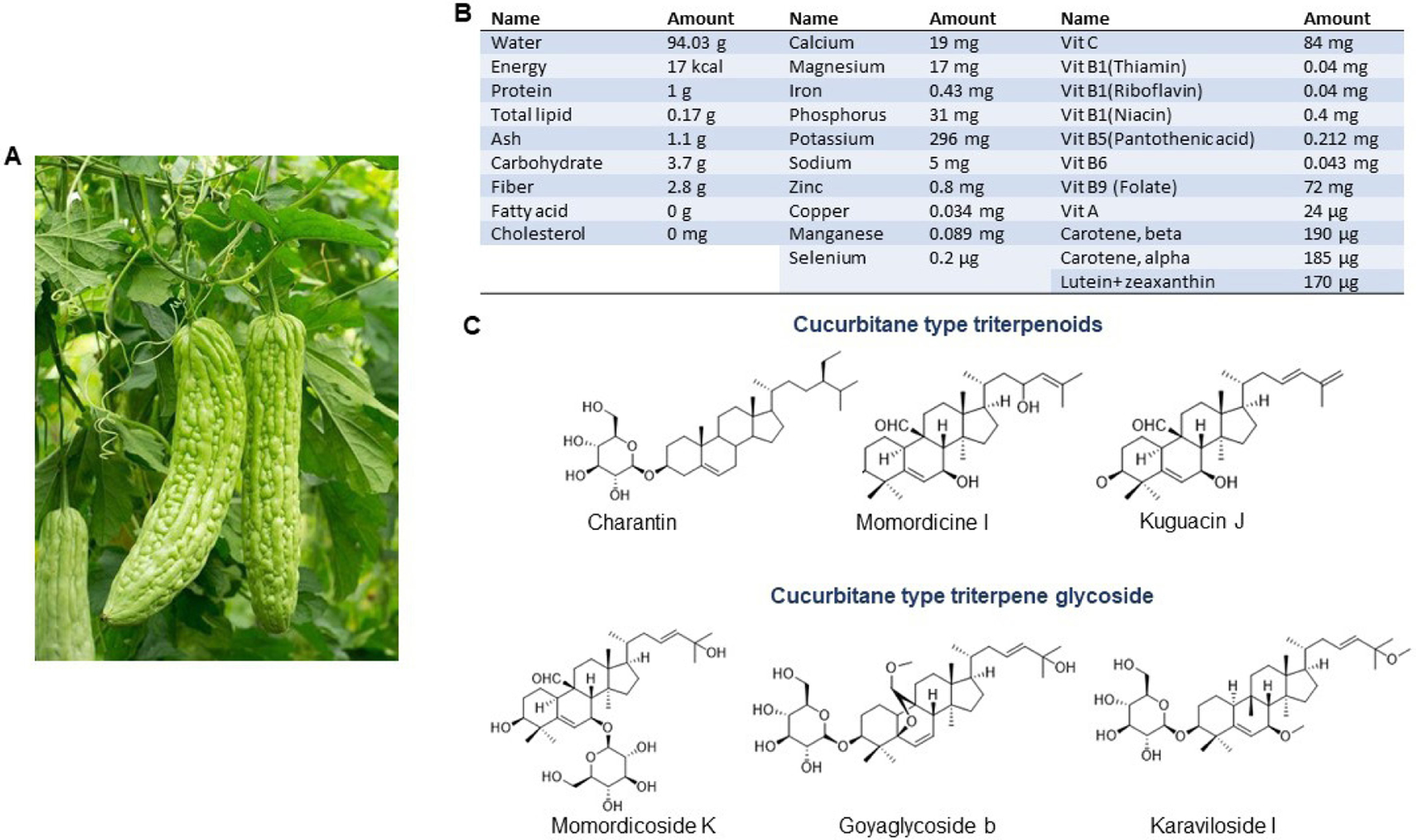
Bitter melon and its constituents. A: Bitter melon vine and its fruit; B: Nutritive substances in bitter melon per 100 g. (Source: U.S. DEPARTMENT OF AGRICULTURE; FDC ID: 168393; NDB Number:11024; FDC: Published:4/1/2019); C: Chemical structure of some major cucurbitane-type triterpenoid and cucurbitane-type triterpene glycoside components.

Bitter melon is a herbaceous plant and is characterized by thin stems, tendrils, bright yellow flowers and light green to yellow fruits [Figure 1A]. It is bitter to the taste but sweet to health as its nutritional value is highest among cucurbits, and thus, the plant has been used in folk medicine since the times of ancient civilization. It is a good source of carbohydrate, protein, fiber, vitamins (vit-C, A, B-complex), minerals (potassium, calcium, zinc, magnesium, phosphorous and iron) and free amino acids (aspartic acid, serine, glutamic acid, threonine, alanine, γ-amino butyric acid)^[24,25,27,28]^ [Figure 1B]. Moreover, it is a plentiful source of phytochemicals, most of which have potential biological activities. The major chemical constituents are classified as cucurbitane-type triterpenoids, cucurbitane-type triterpene glycosides, phenolic acids, flavonoids, essential oils, fatty acids, amino acids, sterols, saponins and proteins; among those, the cucurbitane-type triterpenoids are the most prevalent^[24,28]^. The cucurbitane-type triterpenoids (momordicines I and II) and triterpene glycosides (momordicosides K and L) contribute to the bitterness of the plant^[25]^. Charantin, momordicine I, II and III, karavilagenin A, B, C, D and E, and kuguacins A-S are major cucurbitane-type triterpenoid components [Figure 1C]. The cucurbitane-type triterpene glycosides include momordicosides (A-E, F1, F2, G, I, K, L, M, N, O, Q, R, S and T), charantosides I-VIII, karavilosides (I-XI), goyaglycoside- (a-h), kuguaglycoside, *etc*, [Figure 1C]. Gallic acid, tannic acid, (+)-catechin, caffeic acid, p-coumaric, gentisic acid, chlorogenic acid, and epicatechin are some of the phenolic acids and flavonoids. Palmitic acid and oleic acid are major components of fatty acids with trace constituents such as stearic acid, lauric acid, linoleic acid, arachidic acid, myristic acid and capric acids. Momordica antiviral protein 30 kD (MAP30), α-and β-momocharin, MC2, and marmorins may have potential biological activities.

## BITTER MELON AND ORAL CANCER

**Figure 2.**
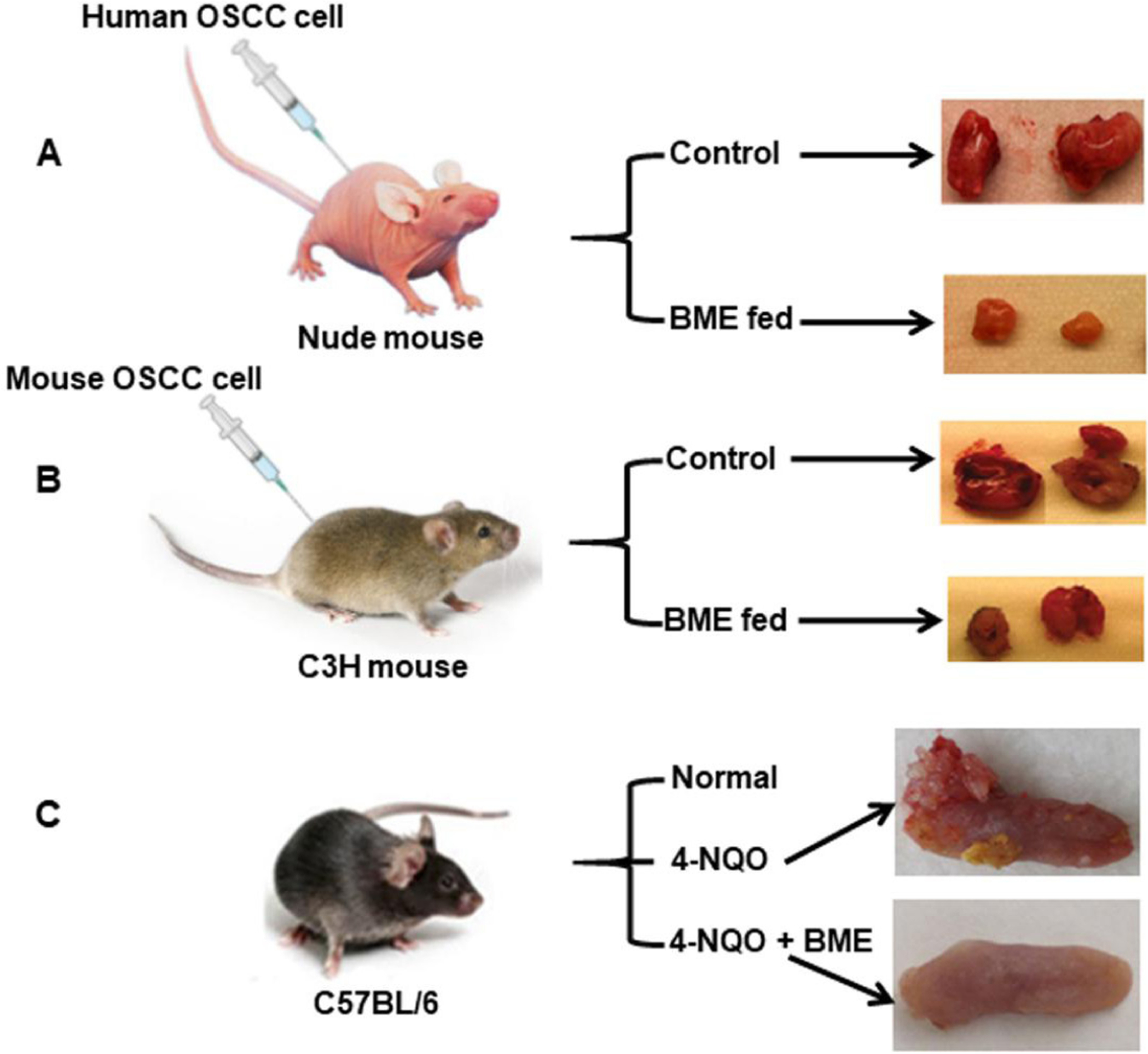
Effect of bitter melon on pre-clinical oral cancer models. A: Xenograft model where human OSCC Cal27 cells were implanted subcutaneously into the flank of athymic nude mice. The mice were divided into a control group and a BME-fed group after palpable tumor formation. Representative images show regression of tumor growth in the BME-fed group^[29]^; B: Syngeneic model where SCCVII cells were injected subcutaneously into the flank of immune-competent mice. Representative images show inhibition of tumor growth following BME treatment through oral gavage^[30]^; C: Immune-competent mice were given a tobacco-associated carcinogen, either 4-NQO (50 μg/mL) alone or both 4-NQO and BME through drinking water. After the 22nd week, invasive tongue squamous cell carcinomas were noted in the cancer group, whereas there was no change in the BME-fed group^[35]^.

The anticancer properties of bitter melon on oral cancer were studied in *in vitro* and *in vivo* models. The aqueous extract of the fruit inhibited head and neck cancer cells Cal27, JHU-29 (JHU029) and JHU-22 (JHU022) in a time-dependent manner^[29]^. The 50% inhibition of cell growth was achieved with 1%−2% doses of a 0.1 g/mL stock for 24 h in all the cells tested. In pre-clinical oral cancer models, the efficacy of the extract was further evaluated [Figure 2]. Oral gavage of BME (100 μL from a 0.1 g/mL stock per mouse) for 4 weeks caused significant regression of Cal27 xenograft tumor growth in nude mice as compared to control mice [Figure 2A]^[29]^. Furthermore, the same dose was found to be similarly effective in the presence of an intact immune system in a syngeneic model [Figure 2B]^[30]^. Mouse squamous cell carcinoma SCCVII cells were implanted into the flanks of C3H mice, and after palpable tumor formation, BME was given by oral gavage. Likewise, BME treatment significantly reduced tumor size and volume in the experimental group as compared to the control mice [Figure 2B]^[30]^. SCCVII cells are a currently available syngeneic model of OSCC, although these cells are derived from a spontaneously formed squamous cell carcinoma in C3H mice^[31–34]^. In addition, a whole exome sequencing study revealed remarkable similarities between SCCVII and oral cancer patient tumor^[34]^. One of the main causative agents of OSCC is the tobacco habit and tobacco-associated carcinogen 4-nitroquinoline 1-oxide (4-NQO) induces invasive tongue squamous cell carcinoma by the 22nd week in immune-competent mice^[35]^. Regular treatment of BME through drinking water significantly reduced tongue cancer incidence with no significant change in normal histology [Figure 2C]^[35]^. In all the animal models, no apparent toxic effects were seen following BME treatment. Several bitter melon compounds have been identified; however, the role of these compounds has not been well evaluated against oral cancer. Some of the compounds showed a cytotoxic effect on laryngeal carcinoma cells (HEp-2) and nasopharyngeal carcinoma cells (CNE-1 and HONE1) of head and neck cancer^[27,36,37]^.

## ORAL CANCER PREVENTION BY BITTER MELON: UNDERLYING MECHANISMS

Molecular mechanisms of bitter melon in prevention of OSCC are summarized below.

### Cell cycle modulation

Deregulation of the cell cycle is one of the major events in OSCC^[38,39]^. This is achieved by frequent modulation of mitogenic and anti-mitogenic response regulatory proteins such as cyclins, cyclin-dependent kinases (CDKs), CDK inhibitors [p21 (WAF1/CIP1), p27 (KIP1), p16 (INK4a)], and retinoblastoma tumor suppressor protein (RB)^[38]^. A focused transcriptomic array followed by protein level validation showed that BME inhibits the expression of cell cycle inducers cyclin D1 and survivin, and activates cell cycle inhibitors p21 and p27 in OSCC cells^[29]^. Down-regulation of transcription factor E2F, proliferating cell nuclear antigen (PCNA), mini-chromosome maintenance complex component 2 (MCM2), karyopherin subunit alpha 2 (KPNA2) and upregulation of ataxia telangiectasia mutated (ATM) and ataxia telangiectasia and Rad3-related protein (ATR) are also evident following BME treatment. Similar effects were also reported in breast and prostate cancer prevention by BME resulting in either S or G2-M phase arrest in the cell cycle^[40,41]^. Thus, cell cycle modulation is an important event in the prevention of oral cancer by BME.

### Modulation in cell signaling

**Figure 3.**
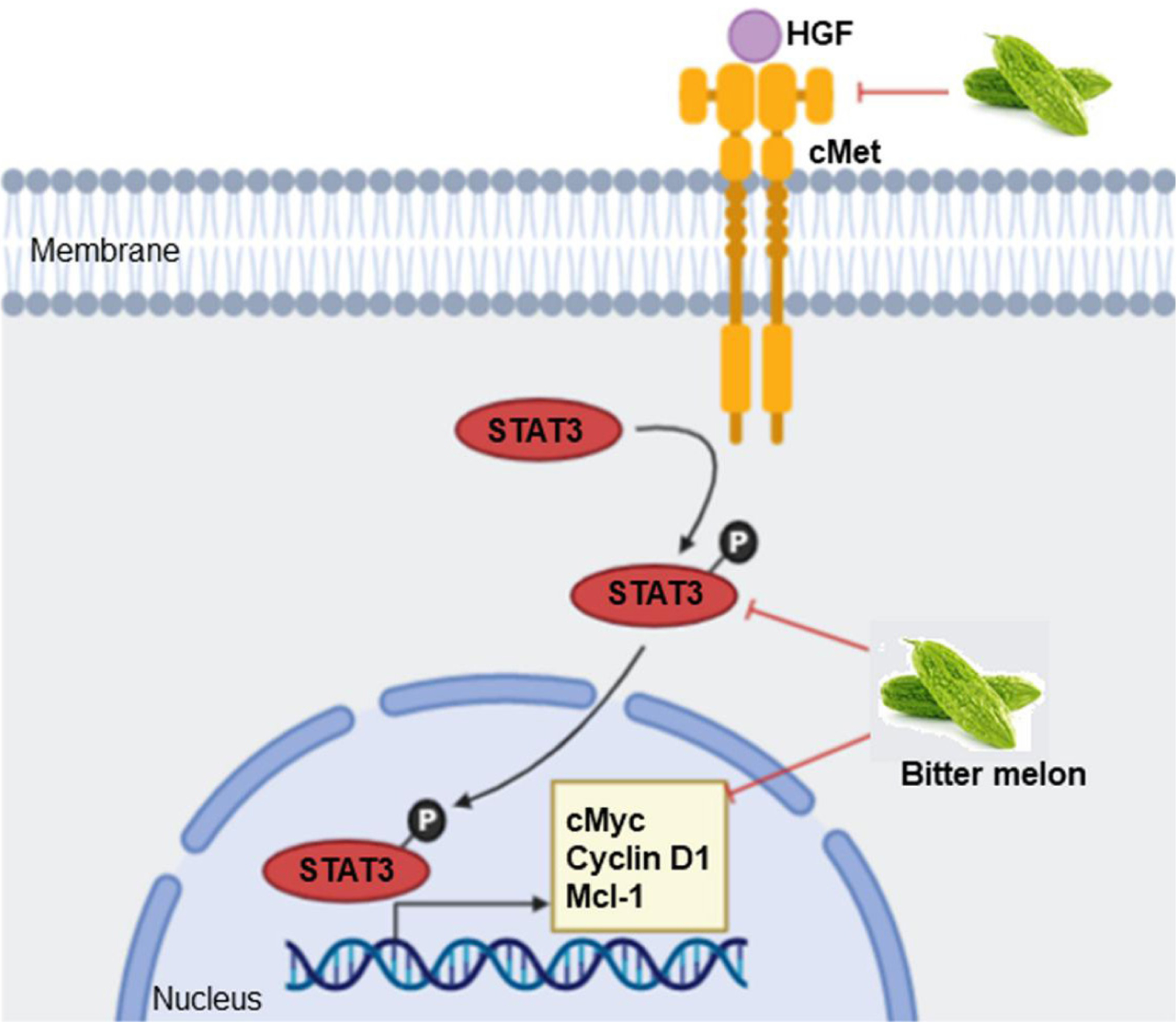
Mode of action of BME in modulating c-Met signaling in oral cancer prevention. BME treatment inhibited expression of key genes of c-Met signaling, resulting in inhibition of cell proliferation and survival. Sharp arrow: Activation/induction; blunt arrow: inhibition.

Alteration in signaling events favor unregulated proliferation, motility and survival of cancer cells^[42]^. Many receptor-ligand signaling events have been investigated in oral cancer, and some of those represent potential targets for cancer therapy. BME treatment inhibited the expression of some key regulatory genes of c-Met (MET proto-oncogene) signaling including the receptor tyrosine kinase *c-Met*, signal transducer and activator of transcription 3 (*STAT3*), *c-Myc* proto-oncogene and myeloid cell leukemia-1 (Mcl-1) in pre-clinical models of OSCC [Figure 3]^[29]^. c-Met regulates multiple downstream signaling including STAT3/c-Myc/cyclin D1 [Figure 3], phosphatidylinositol 3-kinase (PI3K)/AKT serine/threonine kinase (AKT), Ras/mitogen- activated protein kinase (MAPK), Janus kinase (JAK)/signal transducer and activator of transcription (STAT) and Wnt/β-catenin to induce tumor cell proliferation and survival^[43]^. Up-regulation of c-Met signaling is seen in oral cancer, which contributes to treatment resistance to epidermal growth factor receptor (EGFR)-targeting therapies^[44]^. Small molecule inhibitors that target c-Met such as crizotinib, capmatinib, golvatinib, foretinib and the monoclonal antibody ficlatuzumab show promising results either alone or in combination with conventional therapies in OSCC *in vitro* and *in vivo* systems, and some of those are in phase I-II clinical trials^[44]^. However, how BME inhibits the signaling is not known and needs to be evaluated. Modulation in other signaling events such as mechanistic target of rapamycin kinase (mTOR)/p70S6K, AMP-activated protein kinase (AMPK), PI3K/AKT and p38- MAPK signaling are reported in other cancer models following treatment with BME or pure compounds^[26]^.

### Induction of apoptotic cell death

Apoptosis is programmed cell death mediated by caspases and triggered in response to various stimuli such as DNA damage, growth factor withdrawal and oxidative stress^[45]^. Tumor cells often modulate apoptotic molecules in favor of survival. Many studies suggest the importance of apoptosis in response to different anticancer agents, and disruption of this mechanism induces broad drug resistance and sometimes non-specific side effects^[46]^. BME treatment activates effector molecules of apoptosis such as caspases 3 and 9, and it induces poly (ADP-ribose) polymerase (PARP) cleavage in OSCC cells^[29]^. Similarly, BME and pure compounds such as MAP30, RNase MC2, α-, β-momorcharin, 3β,7β,25-trihydroxycucurbita-5,23(E)-dien-19-al, lectin and BG-4 induce apoptosis in other cancer models by inhibiting anti-apoptotic genes and activating pro-apoptotic molecules^[26]^.

### Inhibition of glycolysis and lipid metabolism

**Figure 4.**
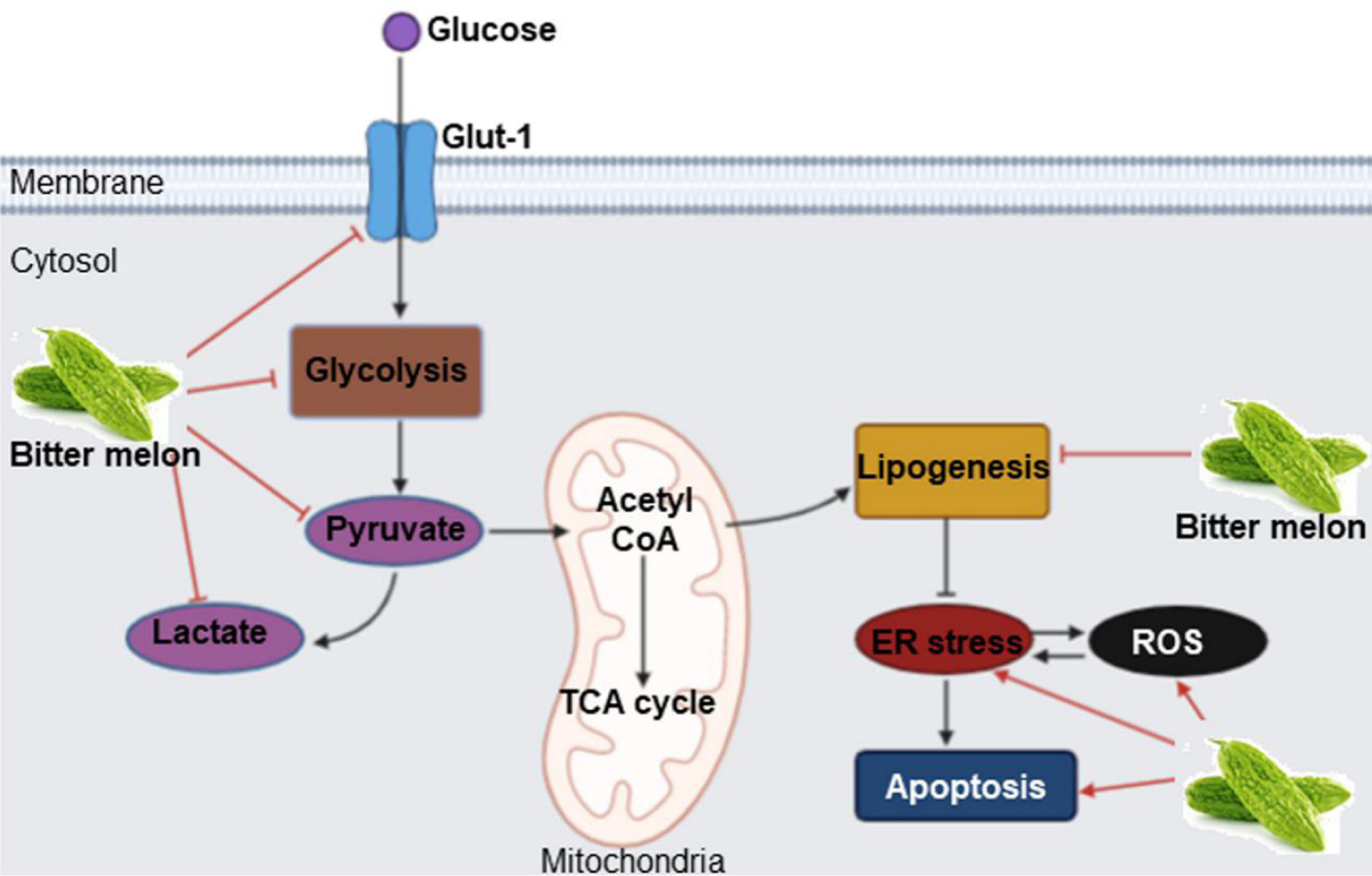
Mode of action of BME in modulating glucose and lipid metabolism in oral cancer prevention. BME treatment inhibits glucose transport and pyruvate and lactate production through glycolysis by inhibiting key genes. BME also inhibits expression of key lipogenesis genes, inducing ER stress and ROS-mediated cell death. Sharp arrow: Activation/induction; blunt arrow: inhibition. The figure is adapted from Sur *et al*.^[47]^, 2019. BME: Bitter melon crude extract; ROS: reactive oxygen species.

Cancer cells modulate their metabolism for rapid energy production, and this reprogramming is one of the hallmarks of cancers. BME treatment inhibits oral cancer metabolism in many ways. Data from RNAseq analysis showed that “metabolic process” is one of the top significantly modulated biological processes in the prevention of 4-NQO-induced mouse tongue cancer by BME^[47]^. Subsequent validation in OSCC cell lines revealed significant reduction of key glycolysis genes such as glucose transporter 1 (GLUT-1)/solute carrier family 2 member 1 (SLC2A1), phosphofructokinase platelet (PFKP), lactate dehydrogenase A (LDHA), pyruvate kinase isozymes M1/M2 (PKM) and pyruvate dehydrogenase kinase 3 (PDK3), resulting in decreased levels of pyruvate and lactate and glycolysis rate [Figure 4]^[47]^. Glucose is an important source of energy and carbon for both normal and cancer cells. However, cancer cells need rapid glucose utilization through glycolysis followed by lactate production even when oxygen is abundant, a phenomenon known as aerobic glycolysis or the Warburg effect^[48]^. Up-regulation of glycolysis is reported in OSCC and inhibition of glycolytic genes have shown promising results in OSCC pre-clinical and clinical systems^[47]^. In case of lipid metabolism, BME inhibited the expression of fatty acid biogenesis genes ATP citrate lyase (ACLY), acetyl-CoA carboxylase 1(ACC1) and fatty acid synthase (FASN) [Figure 4]^[47]^. Enhanced expression of the lipogenic enzymes is universal phenotypic alteration in most tumors and therapeutic targeting of those enzymes shows promising results in pre-clinical systems^[49]^. Lipidomics analysis showed significant reduction in several molecular species of phosphatidylcholine (PC) and phosphatidylethanolamine (PE), specifically plasmenyl-ethanolamine (pPE), which are the most abundant membrane phospholipids in mammalian cells and subcellular organelles, following BME treatment in OSCC cells. Consequently, reduced calcium-independent phospholipase A2 activity, reduced expression of the membrane lipid raft marker flotillins, induction of reactive oxygen species (ROS) and endoplasmic reticulum (ER) stress-induced cell death were evident in OSCC cells following BME treatment [Figure 4]^[47]^. Inhibition of glucose and lipid metabolism was also evident in the prevention of breast and pancreatic cancers by BME^[26]^.

### Induction of anticancer immunity

**Figure 5.**
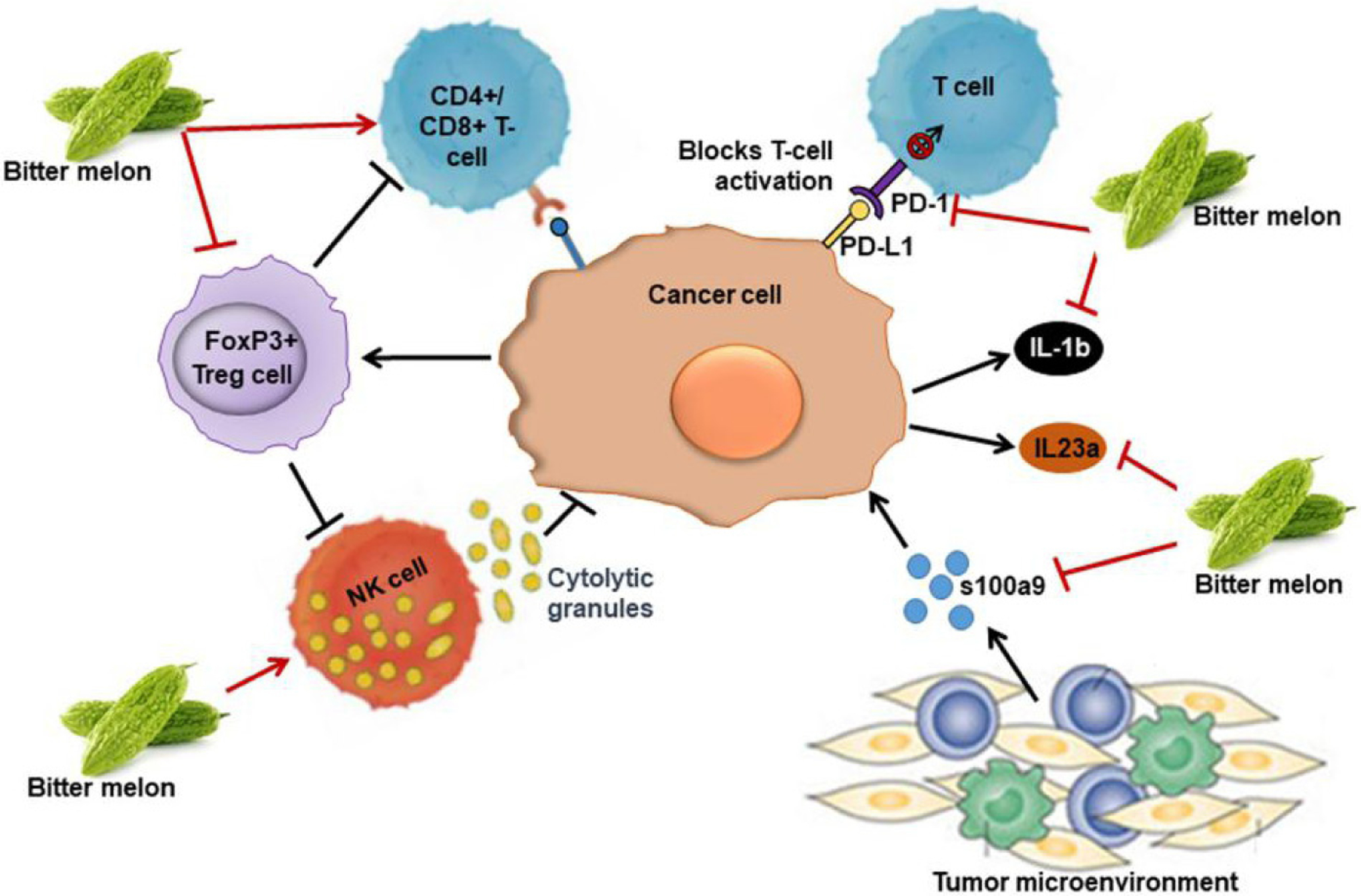
Immune modulatory role of BME in preventing oral cancer. BME treatment induces T cell populations in BME-treated tumors, NK cell-mediated cytotoxic effects and decreases FoxP3+ regulatory T cells (Treg) cell population. It also inhibits pro-inflammatory molecules s100a9, interleukin 23 subunit alpha (IL23a), interleukin 1 beta (IL1β) and immune checkpoint gene *PDCD1* (*PD1*), inducing antitumor immunity.

A degree of host immune suppression is evident in the progression of cancer, and it is a big challenge for cancer therapy. Currently, many immune therapy drugs are approved for the treatment of many cancers. The immune modulatory role of bitter melon is seen in the prevention of oral cancer. In an *in vivo* syngeneic model, BME treatment inhibited mouse tumor growth^[30]^. Further evaluation of the tumor and spleen tissues revealed that there was significant induction of CD4+ and CD8+ T cell populations in the BME-fed group^[30]^. In addition, BME treatment reduced the Th17 cell population in the tumor; while the Th1 and Th2 cell populations were unchanged. On the other hand, the regulatory T cells (Treg) (CD4+CD25+FoxP3+) cell population was significantly reduced in the spleen and tumor of the BME-fed mice, suggesting its immunomodulatory role [Figure 5]. Increased Treg population is seen in tumors, which is responsible for immune suppression. Unlike T cells, natural killer (NK) cells respond quickly and thus play an important role in the innate immune response. NK cells recognize foreign cells, including tumor cells, by surface molecules such as MHC-I and kill the target cells by releasing cytokines and cytolytic granules containing perforin and granzyme B. BME treatment enhances NK cell-mediated cytotoxic effects on OSCC cells when they are co-cultured with BME-treated NK cells [Figure 5]^[50]^. No cytotoxic effect of BME alone was observed on NK cells, but BME did induce granzyme B accumulation, translocation/accumulation of CD107a/LAMP1 and expression of *CD16* and *NKp30*^[50]^. Furthermore, RNAseq analysis indicated significant modulation of “immune system processes” in the prevention of mouse tongue carcinogenesis by BME^[35]^. The extract significantly reduces the expression of pro-inflammatory molecules *s100a9*, interleukin 23 subunit alpha (IL23a), interleukin 1 beta (IL1β) and immune checkpoint gene *PDCD1*/programmed cell death-1 (*PD1*) during tongue cancer prevention [Figure 5]. But how BME inhibits the immune system molecules is not known and needs to be evaluated in future studies. Increased expression of the pro-inflammatory genes has been demonstrated in human malignancies including OSCC^[35,51]^. S100A9 has both tumor supportive and tumor suppressive roles^[52,53]^. We and others have observed the high expression of *s100a9* in 4-NQO-induced mouse oral and esophageal carcinogenesis^[35,54]^. On the other hand, elevated expression of *s100a9* was reported in oral cancer patient tumors and serum samples at early stages^[51]^. The molecular mechanism behind the dual role of s100a9 in OSCC samples is unknown and needs further investigation. Inhibitors of s100a9 or PD1 exhibited promising outcomes in phase I-III clinical trials against different cancers^[52,55,56]^. Thus, immune modulation is an important role of bitter melon in preventing OSCC.

## CONCLUSION

Bitter melon is a medicinal plant and a good foodstuff. Several pre-clinical studies show its potential anticancer role. Nowadays, many studies suggest the use of alternative therapies and plant-based medicines either alone or in combination with conventional therapy for better management of the disease. Other advantages of the natural phytochemicals are absence of toxic side effects and cost effectiveness. Many phytochemicals are already in clinical trials. In this review, we mainly focused on the effect of bitter melon in preventing oral cancer. Bitter melon is a good source of nutrients and active components which include triterpenoids, triterpene glycosides, phenolic acids, flavonoids, lectins, sterols, proteins and saponins. The crude extract is highly effective in killing oral cancer cells and preventing tumor growth in mice. The extract inhibits the cell cycle and cell signaling, induces apoptotic cell death, inhibits glucose and lipid metabolism in cancer cells and most importantly modulates the immune system to prevent oral cancer. In animal models, no toxic effects of the extract are reported so far. Thus, it seems that regular intake of bitter melon or bitter melon juice will be good to boost the body’s immune defence system as well as to prevent oral cancer risk. On the other hand, the extract may be used as a therapeutic agent along with current standard therapy for better management of OSCC. Further mechanistic evaluation of active components in pre-clinical systems is required for designing prospective studies for intervention trials.
